# Common and Rare Variants Genetic Association Analysis of Circulating Neutrophil Extracellular Traps

**DOI:** 10.3389/fimmu.2021.615527

**Published:** 2021-02-24

**Authors:** Samantha J. Donkel, Eliana Portilla Fernández, Shahzad Ahmad, Fernando Rivadeneira, Frank J. A. van Rooij, M. Arfan Ikram, Frank W. G. Leebeek, Moniek P. M. de Maat, Mohsen Ghanbari

**Affiliations:** ^1^ Department of Hematology, Erasmus University Medical Center, Rotterdam, Netherlands; ^2^ Department of Epidemiology, Erasmus University Medical Center, Rotterdam, Netherlands; ^3^ Division of Systems Biomedicine and Pharmacology, Leiden Academic Centre for Drug Research, Leiden University, Leiden, Netherlands; ^4^ Department of Internal Medicine, Erasmus University Medical Center, Rotterdam, Netherlands

**Keywords:** genome-wide association studies, genetics, exome sequencing, neutrophil extracellular traps, NETs

## Abstract

**Introduction:**

Neutrophils contribute to host defense through different mechanisms, including the formation of neutrophil extracellular traps (NETs). The genetic background and underlying mechanisms contributing to NET formation remain unclear.

**Materials and Methods:**

We performed a genome-wide association study (GWAS) and exome-sequencing analysis to identify common and rare genetic variants associated with plasma myeloperoxidase (MPO)-DNA complex levels, a biomarker for NETs, in the population-based Rotterdam Study cohort. GWAS was performed using haplotype reference consortium(HRC)-imputed genotypes of common variants in 3,514 individuals from the first and 2,076 individuals from the second cohort of the Rotterdam Study. We additionally performed exome-sequencing analysis in 960 individuals to investigate rare variants in candidate genes.

**Results:**

The GWAS yielded suggestive associations (p-value < 5.0 × 10^−6^) of SNPs annotated to four genes. In the exome-sequencing analysis, a variant in *TMPRSS13* gene was significantly associated with MPO-DNA complex levels (p-value < 3.06×10^−8^). Moreover, gene-based analysis showed ten genes (*OR10H1, RP11-461L13.5, RP11-24B19.4, RP11-461L13.3, KHDRBS1, ZNF200, RP11-395I6.1, RP11-696P8.2, RGPD1, AC007036.5*) to be associated with MPO-DNA complex levels (p-value between 4.48 × 10^−9^ and 1.05 × 10^−6^). Pathway analysis of the identified genes showed their involvement in cellular development, molecular transport, RNA trafficking, cell-to-cell signaling and interaction, cellular growth and proliferation. Cancer was the top disease linked to the NET-associated genes.

**Conclusion:**

In this first GWAS and exome-sequencing analysis of NETs levels, we found several genes that were associated with NETs. The precise mechanism of how these genes may contribute to neutrophil function or the formation of NETs remains unclear and should be further investigated in experimental studies.

## Introduction

Neutrophils play important roles in host defense through different mechanisms, including the formation of neutrophil extracellular traps (NETs) (1). NETs are released as a result of a unique form of cell death, where DNA coated with histones and anti-microbial proteins, such as neutrophil elastase and myeloperoxidase (MPO), form a web-like structure (1). Bacteria, fungi, viruses and parasites have been shown to induce NET formation, where they are trapped and killed by NETs, preventing dissemination of pathogens (1). Whereas NETs have a protective role in host defense against pathogens, NETs have also been shown to be involved in the pathogenesis of various diseases including, thrombosis (2), cardiovascular diseases (3, 4), auto-immune diseases (5) and sepsis (6). Although the role of NETs in health and disease has been postulated, the molecular mechanisms of NET formation remain elusive.

Genome-wide association studies (GWAS) and exome-sequencing analysis have been successfully implemented as approaches to identify genetic variants associated with disease susceptibility. The assessment of genetic variants in association with NETs might help to elucidate potential molecular mechanisms intervening in their formation and their downstream effect on other pathways. Here, we are the first to apply these approaches to ascertain common and rare genetic variants associated with NETs using data from a population-based cohort study. We performed additional in silico analyses to identify more evidence for the associated variants and genes in relation to the plasma MPO-DNA complex levels.

## Materials and Methods

### Study Description and Population

The Rotterdam Study (RS) is a prospective, population-based cohort study of determinants of chronic diseases in older adults (7). The first cohort (RS-I) started in 1990 and included 7,983 inhabitants, aged ≥55 years from Ommoord, a suburb of Rotterdam in the Netherlands. The cohort was further extended in 2000 (RS-II) and 2005 (RS-III), establishing a total of 14,926 participants. Every 4 years participants are interviewed at their homes and they visit the study center for an extensive clinical assessment, including venipuncture and assessment of various cardiometabolic risk factors. The RS was approved by the Medical Ethics Committee of the Erasmus MC and by the Ministry of Health, Welfare and Sport of Netherlands. All participants provided written informed consent to participate in the study in accordance with the Declaration of Helsinki.

### MPO-DNA Complex Measurement

Citrated plasma samples were collected at the third visit of RS-I (1997–1999) and the baseline examination of RS-II (2000–2001), and stored at −80°C. We determined circulating levels of NETs by measuring myeloperoxidase (MPO)-DNA complexes with a capture ELISA as reported earlier (4). Briefly, we adjusted the commercial human cell death ELISA kit (Cell death detection ELISAPLUS, Cat. No 11-774-425-002; Roche Diagnostics Nederland B.V., Almere, the Netherlands) in which we used anti-MPO monoclonal antibody (clone 4A4, ABD Serotec, # 0400-002) as the capturing antibody. Patient plasma was added together with the peroxidase-labeled anti-DNA monoclonal antibody (component No.2 of the commercial cell death detection ELISA kit; Roche, #11-774-425-002). The absorbance at 405 nm wavelength was measured using BioTek Synergy HT plate reader with a reference filter of 490 nm. Values are expressed as milli arbitrary units (mAU/ml). The reference line to define the mAU was composed after isolation and induction of neutrophils from a healthy donor. NET formation was induced by adding phorbol 12-myristaat 13-acetaat (PMA).

### Genotyping and Imputation

**Figure 1:**
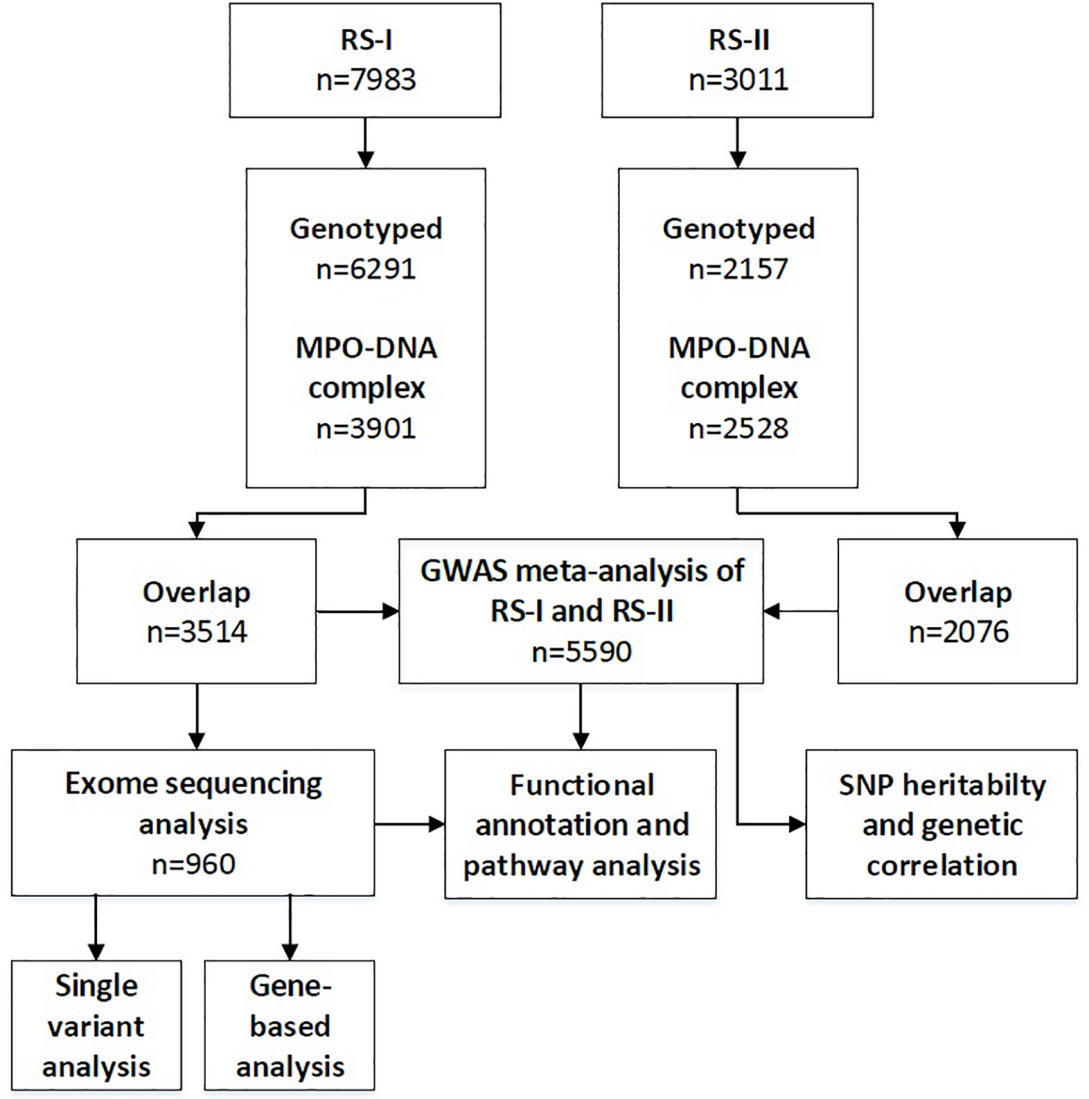
Flow diagram of the number of included individuals and performed analyses. RS-I, Rotterdam Study cohort 1; RS-II, Rotterdam Study cohort 2; GWAS, genome-wide association studies; SNP, single-nucleotide polymorphism.

We implemented two different genetic association studies covering the assessment of common variants and rare variants (**Figure 1**). Genomic DNA was extracted from peripheral blood mononuclear cells. Genome-wide single-nucleotide polymorphisms (SNPs) were genotyped from 6,291 participants from RS-I and 2,157 participants from RS-II using the Illumina Infinium II HumanHap550 or 610quad arrays. All genotyped participants were of European ancestry based on their self-report. Before imputation, genotyped SNPs with a call rate of < 98%, a minor allele frequency (MAF) of < 1%, or a Hardy–Weinberg equilibrium p-value of < 1 × 10^−6^, were excluded. In RS-I, a total of 512,849 SNPs remained after filtering and these were used for imputation. In RS-II, a total of 537,405 SNPs were used for imputation. Dosages of 19,537,258 SNPs were imputed in both studies using the Haplotype Reference Consortium (HRC), a reference panel of 64,976 human haplotypes at 39,235,157 SNPs constructed using whole-genome sequence data from 20 studies of predominantly European ancestry (8). The imputation was conducted using the Michigan Imputation server (9). The server uses SHAPEIT2 (v2.r790) to phase the data and Minimac 4 for imputation to the HRC reference panel (v1.1). After imputation, SNPs with a MAF < 0.01 or an imputation quality < 0.3 were excluded. The overlap between participants with MPO-DNA complex measurements and genotypes was 3,515 in RS-I and 2,076 in RS-II (**Figure 1**).

Whole-Exome sequencing was conducted at the Human Genomics Facility in Erasmus Medical Centre, Rotterdam (10). Exome-SNPs were genotyped using Illumina HumanExome BeadChip v1.0 and was conducted on 2,998 paired-end sequenced samples using the Illumina hiSeq2000 (2×100bp reads). Indels and single nucleotide variants were filtered out and evaluated using GAKs Variant Evaluation; variants with a call rate < 0.97, duplicate samples, duplicate variants and > 5% of missing genotypes were considered as exclusion criteria. A total of 2,628 samples passed through all technical quality control and GATKs Haplotype Caller was used to call SNPs and indels simultaneously. Genotype calling was performed according to the CHARGE joint calling protocols (11). Annovar software tool (12) was used to functionally annotate each genetic variant. Each variant was coded as 0, 1 and 2 representing two reference alleles, one reference allele and one mutated allele and two mutated alleles, respectively.

### Common Variant Analysis

GWAS were carried out in RS-I and RS-II under the additive model using HRC-imputed data and RVtest tool (13). Linear regression was modeled using log-transformed MPO-DNA complex levels with adjustment for age, sex, and the first four principal components. EasyQC was used to conduct quality control across cohorts (excluded: MAF < 0.01) to identify file naming errors, erroneous SNP genotype data and false association caused by incorrect analysis models (14). Cleaned results were combined in a joint meta-analysis of linear regression estimates, and standard errors using an inverse-variance weighting approach was conducted using METAL (15). We determined the association of each SNP with MPO-DNA complex as the regression slope, its standard error and its corresponding p-value. Variants with a meta-analyzed p-value < 5.0 ×10^−8^ were considered significant. The threshold for suggestive associations was set at a p-value < 5.0 ×10^−6^.

### Gene Set Enrichment Analysis

A gene-set analysis of GWAS data was done using MAGMA v1.06 (16), implemented by FUMA v1.3.2 (17). Gene-based approach aims to test the joint association of all markers in the gene with MPO-DNA complex. Gene-set analysis aggregates individual genes to groups of genes sharing certain biological, or other functional characteristics, allowing the identification of effects consisting of multiple weaker associations to determine their joint effect. Likewise, gene-set analysis might provide insight into the involvement of specific biological pathways or cellular functions underlying NET formation (16). The analysis was performed using summary-level meta-analysis results. First, a gene-based association analysis was done to identify candidate genes associated with MPO-DNA complex. Second, the genes identified from the gene-based analysis were used to perform a tissue enrichment analysis using gene expression data for 53 tissues from GTEx. For all MAGMA analyses, multiple testing was accounted for Bonferroni correction.

### Rare Variant Analysis

Exome-sequencing analysis was performed in a subset of RS-I subjects (N=960) to evaluate the association between rare variants and MPO-DNA complex levels. We used log-transformed MPO-DNA complex levels with adjustment for age, sex, BMI, smoking, SBP, cholesterol, HDL, and four ancestry-informative principal components (PCs), because rare variants are more susceptible to population stratification (18). Estimated regression coefficient for each variant and its standard error (prepScores) were employed on single variant level, to perform score tests for single SNP associations and gene-based test [sequence kernel association test (SKAT)] using the seqMeta package implemented in R (19). SKAT is a bidirectional test and is more powerful when the effect direction of rare variants within a gene varies. Single-variant analysis was done using score tests. We defined a statistical significance threshold of single variant and gene-based exome sequencing based on Bonferroni correction for multiple testing, ~606, 583 variants (p-value < 8.2 × 10^−8^) and ~46,551 genes (p-value < 1.07 × 10^−6^), respectively.

### SNP Heritability and Genetic Correlation

To characterize the extent to which common genetic variants determine MPO-DNA complex levels, and shared genetic etiology with other traits (coronary artery disease, stroke and C-reactive protein), we applied linkage disequilibrium score regression (LDSC) (20) methods for estimating SNP heritability and genetic correlation based on genome-wide sharing between distantly related individuals. LDSC is a summary-statistics-based method which estimates heritability and genetic correlation while accounting for LD and requires only publicly available summary statistics from genetic studies (21). In brief, the cross-product of two GWAS test statistics is calculated at each genetic variant, and this cross-product is regressed on the LD score. The slope of the regression is used for estimating the genetic covariance between two phenotypes. We used the default European LD score file based on the European 1KG reference panel. The analyses were conducted using LDSC (LD SCore) v1.0.1 package running under R (https://github.com/bulik/ldsc) (20, 22).

### Functional Annotation and Pathway Analysis

We used HaploReg v4.1 (http://www.broadinstitute.org/mammals/haploreg/haploreg.php; in the public domain) (23) to retrieve proxy SNPs in high linkage disequilibrium (LD) (*R* ^2^ threshold > 0.8, limit distance 100 kb, and population panel CEU) with the associated (suggestive) variants and subsequently to identify the SNP position and effect on protein structure, gene regulation, and splicing. Moreover, the SNPs in LD were scrutinized in the GWAS catalog to ascertain possible association with other traits (24). Additionally, to assess the correlation between the identified SNPs and expression levels of the host transcripts, we used expression quantitative trait loci (cis-eQTL) data from the Genotype-Tissue Expression Project (GTEx portal, Analysis Release V7) (http://www.gtexportal.org/home/; in the public domain) and from 3,841 whole blood samples from five Dutch biobanks (BIOS-BBMRI database) (http://www.genenetwork.nl/biosqtlbrowser/; in the public domain). GTEx is a platform with available expression data on potential target organs (heart tissue, kidney tissue, brain tissue, aortic endothelial cells, and blood vessels) as well as blood cell types (CD4+ macrophages, monocytes) (25). The gene expression values are shown in TPM (transcripts per million), calculated from a gene model with isoforms collapsed to a single gene. Box plots are shown as median and 25^th^ and 75^th^ percentiles, outliers are displayed as dots if they are above or below 1.5 times the interquartile range (25).

We used GeneMANIA Cytoscape plugin to identify the most related genes to a query gene set using a guilt-by-association approach. The platform employs a large database of functional interaction networks from multiple organisms and each related gene is traceable to the source network used to make the prediction (26).We additionally conducted a core analysis, implemented in QIAGEN’s Ingenuity Pathway Analysis Software (IPA, http://www.ingenuity.com), to determine enriched pathways and to interpret the role of these genes in the context of biological processes, pathways and molecular networks. IPA is a knowledge database generated from peer-reviewed scientific publications that enables the discovery of highly represented biological mechanisms, pathways or functions most relevant to the genes of interest from large, quantitative datasets. We uploaded all target genes and performed a core IPA analysis with default setting. IPA uses right-tailed Fisher exact test to identify enriched canonical pathways and diseases associated to these genes.

## Results

### Characteristics of Study Participants

The baseline characteristics of participants from RS-I and RS-II included in the GWAS and exome-sequencing analysis are shown in **Table 1**. Data on GWAS and MPO-DNA complex levels were available in 3,514 participants of RS-I and in 2,076 participants of RS-II. The mean age of all the participants was 68.8 ± 8.5 years and the majority of the study participants were females (56.6%). Participants in RS-I were older (mean age 72.5 ± 7 years) than participants in RS-II (mean age, 64.9 ± 8 years). The median MPO-DNA complex level in RS-I was 52 mAU/ml (25^th^ to 75^th^ percentile 41-82) and 56 mAU/ml (46-93) in RS-II. Moreover, exome sequencing analysis for MPO-DNA complex levels was performed in 960 participants from RS-I. The mean age of these participants was 70.7 ± 6.1 years and the majority of the subjects were females (57.1%). Median MPO-DNA complex levels were 53 mAU/ml (41-79).

**Table 1:** Characteristics of RS-I and RS-II participants included in this study.

	RS-I (GWAS)	RS-I (Exome sequencing)	RS-II (GWAS)
Sample size (n)	3514	960	2076
Mean age ± SD	72.5 ± 7	70.7 ± 6.1	64.9 ± 8
Male sex (n, %)	1475 (42)	412 (43)	949 (45.6)
Median MPO-DNA complex (mAU/ml), 25^th^–75^th^ percentile	52 (41–82)	53 (41–79)	56 (46–93)
Mean ln MPO-DNA complex ± SD	4.1 ± 0.7	4.1 ± 0.7	4.2 ± 0.7
BMI (kg/m^2^)	26.8 ± 3.9	26.8 ± 3.7	27.2 ± 4.0
Current smoking (n, %)	607 (15.6)	147 (15.3)	409 (19.7)
Total cholesterol (mmol/L) ± SD	5.8 ± 1.0	5.8 ± 0.9	5.8 ± 1.0
HDL cholesterol (mmol/L) ± SD	1.4 ± 0.4	1.4 ± 0.4	1.4 ± 0.4
Systolic blood pressure (mm Hg) ± SD	143.4 ± 21.4	143 ± 20.4	143.2 ± 21.3
Diastolic blood pressure (mm Hg) ± SD	76 ± 11.2	76 ± 11	79 ± 11.0
Prevalent type 2 diabetes (n, %)	451 (11.5)	97 (10)	264 (12.7)

Data are presented for RS-I, the subset of participants of RS-I were exome sequencing was performed and RS-II, separately. MPO-DNA complex, myeloperoxidase-DNA complex; BMI, body mass index; HDL, high-density lipoprotein.

### Association Study With Common Variants

We first performed a GWAS in each RS cohort. Fixed-effect meta-analysis of the summary statistics from RS-I and RS-II showed no significant associations between common variants and MPO-DNA complex levels passing the GWAS threshold of 5.0 × 10^−8^ (**Supplementary Table S1**). The lowest p-value was found for rs289078 located near a long intergenic non-protein coding RNA 2501 (*LINC02501*; chr4:31416764). The minor allele T at this region was associated with a decrease in MPO-DNA complex levels (β= 0.1, p-value= 3.5 × 10^−7^). We additionally queried association results between rs289078 and over 778 traits assessed in 452264, as implemented in Gene Atlas (http://geneatlas.roslin.ed.ac.uk/) (27). At nominal significance level, rs289078 was found to be associated with postpartum hemorrhage, deep venous thrombosis, among others (**Supplementary Table S2**).

**Figure 2:**
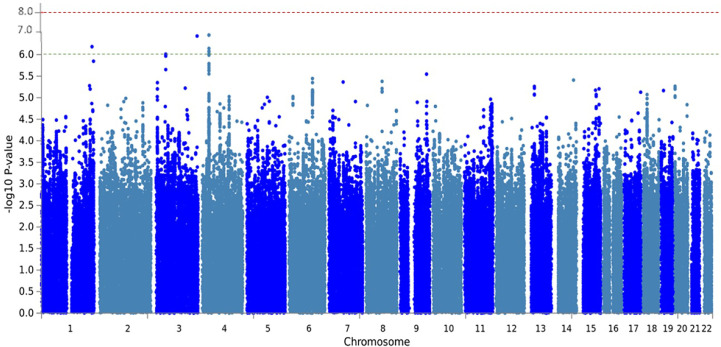
Manhattan plot of meta-analysis GWAS with MPO-DNA levels of RS-I and RS-II. The Manhattan plot shows the statistical genetic association between SNPs and MPO-DNA complex levels. Each SNP is represented by a dot. Genomic coordinates are displayed along the x-axis and the negative log-base-10 of the p-value for each of the polymorphisms in the genome is displayed along the y-axis. The red line represents the genome-wide significance threshold (5 × 10^−8^). The SNPs with a suggestive association with MPO-DNA levels were annotated to the genes*, KIF26B, CDK19, CATSPERB, AC027119.1*.

Moreover, we found 26 additional SNPs, most of them located in intergenic regions. Five of these SNPs were annotated to 4 genes (*KIF26B, CDK19, CATSPERB, AC027119.1*) that were suggestively associated with MPO-DNA complex levels (p-value < 5.0 × 10^−6^) (**Supplementary Table S1**). Manhattan plot depicting the association of all common SNPs with MPO-DNA complex levels is shown in **Figure 2**. QQ-plot is shown in **Supplementary Figure S1**.

### Gene Set Enrichment Analysis

We performed gene-based association analyses using MAGMA to identify tissues and pathways relevant to NET formation. Input SNPs were mapped to 18889 protein coding genes establishing a genome wide significance p-value = 0.05/18884 = 2.6 × 10^−6^. There were no genes associated with MPO-DNA complex levels at genome-wide significance (**Supplementary Table S3**). The lowest p-value was found for a set of 40 SNPs located in *TAS1R1* (Taste 1 Receptor Member 1) (p-value=2.7 × 10^−5^). Tissue specificity analysis for *TAS1R1*, evaluated across 53 tissue types from GTEx project, showed no significant differential expression. Likewise, gene set analysis revealed no significant pathways. Gargalovic response to oxidized phospholipids red up, composed of a set of 15 genes, showed the lowest p-value= 3.2 × 10^−5^ (**Supplementary Table S4**).

### Association Study With Rare Variants

In the single-variant analysis, we found one variant corresponds to chr11:117789326, annotated to the *TMPRSS13* (Transmembrane Serine Protease 13) gene, significantly associated with MPO-DNA complex levels. The minor allele C has was negatively associated with the plasma MPO-DNA complex levels (β= −4.00, p-value= 3.06 × 10^−8^). Furthermore, gene-based analysis with rare variants showed 10 genes (*OR10H1, RP11-461L13.5, RP11-24B19.4, RP11-461L13.3, KHDRBS1, ZNF200, RP11-395I6.1, RP11-696P8.2, RGPD1, AC007036.5)* to be significantly associated with MPO-DNA complex levels, passing the significant threshold of 1.07 × 10^−6^ (**Table 2**). The strongest association was observed for the *OR10H1* (Olfactory Receptor Family 10 Subfamily H Member 1) gene, composed of six rare variants (p-value= 4.48 × 10^−9^) located on chromosome 19. The top variant driving the gene-based statistic was 19:15918484 (p-value = 1.19 × 10^−8^).

**Table 2:** Genes associated with MPO-DNA complex levels through exome-sequencing analysis.

Gene	MAF	Number of SNPs	p-value
*OR10H1*	0.003	6	4.48 × 10^−9^
*RP11-461L13.5*	0.003	3	1.32 × 10^−7^
*RP11-24B19.4*	0.003	3	1.93 × 10^−9^
*RP11-461L13.3*	0.005	6	2.44 × 10^−7^
*KHDRBS1*	0.005	7	3.12 × 10^−7^
*ZNF200*	0.004	5	3.49 × 10^−7^
*RP11-395I6.1*	0.001	3	3.51 × 10^−7^
*RP11-696P8.2*	0.002	4	3.78 × 10^−7^
*RGPD1*	0.014	12	7.58 × 10^−7^
*AC007036.5*	0.002	4	1.05 × 10^−6^
*RPL21^†^*	0.003	6	1.10 × 10^−6^
*SNORA27^†^*	0.003	6	1.10 × 10^−6^
*SNORD102^†^*	0.003	6	1.10 × 10^−6^
*TMEM55B^†^*	0.019	21	1.15 × 10^−6^
*ZDHHC19^†^*	0.008	10	1.52 × 10^−6^
*RTN4R^†^*	0.011	15	1.77 × 10^−6^
*FOXC1^†^*	0.004	5	2.62 × 10^−6^

Genes found through exome sequencing analysis that are associated with MPO-DNA complex levels.

The significance threshold for gene-based analysis set at p-value < 1.07 × 10^−6^. ^†^Genes with a suggestive association with MPO-DNA complex levels. MAF, minor allele frequency; SNP, single-nucleotide polymorphism.

Additionally, we did a look up for association between common variants in the 10 genes identified by exome-sequencing analysis and MPO-DNA complex levels in our GWAS results. A common variant (chr1:32509964) located in *KHDRBS1* (KH domain-containing, RNA-binding, signal transduction-associated protein 1) gene was nominally associated with MPO-DNA complex levels (β= 0.17, p-value=0.003). In addition, we checked for rare variants in the 4 suggestive genes identified by GWAS in our exome-sequencing results, and found no significant association.

### SNP Heritability and Functional Annotation

Our study includes data from 5,590 participants from two European cohorts. The 1KG intercept was 1.02 (standard error= 0.006). The LD-score calculated SNP Heritability showed an estimate of 7% and the mean chi^2^ was below ~1.02 (chi^2^ = 1). Moreover, either sample size or heritability was not sufficient to determine cross-trait genetic correlation.

None of the 27 suggestive SNPs identified by GWAS have nonsynonymous proxy variants in strong LD (*R* ^2^ > 0.8) (**Supplementary Table S5**). We identified 155 proxy SNPs (*R* ^2^ > 0.8) of these GWAS SNPs. We checked GWAS Catalog to see whether the identified variants or their proxies have been reported previously to be associated with any diseases or traits (e.g., cardiovascular disease, Alzheimer’s disease, etc.). We found that none of the SNPs were reported to be associated with any outcome in the GWAS Catalog. We further investigated gene expression levels associated with GWAS-SNPs and their proxies (SNPs in LD), as reported by GTEx. Among these, proxies of rs10457220 and rs112514818, located in *CDK19*, were associated to gene expression levels of their host gene (**Supplementary Tables S6 and S7**). We also investigated gene expression levels across different tissues of the genes identified by exome-sequencing analyses, as reported by GTEx (**Supplementary Figures S2–S7**). Although the expression levels of these genes have not been reported for neutrophils, the data show some of them to be expressed in whole blood.

**Figure 3:**
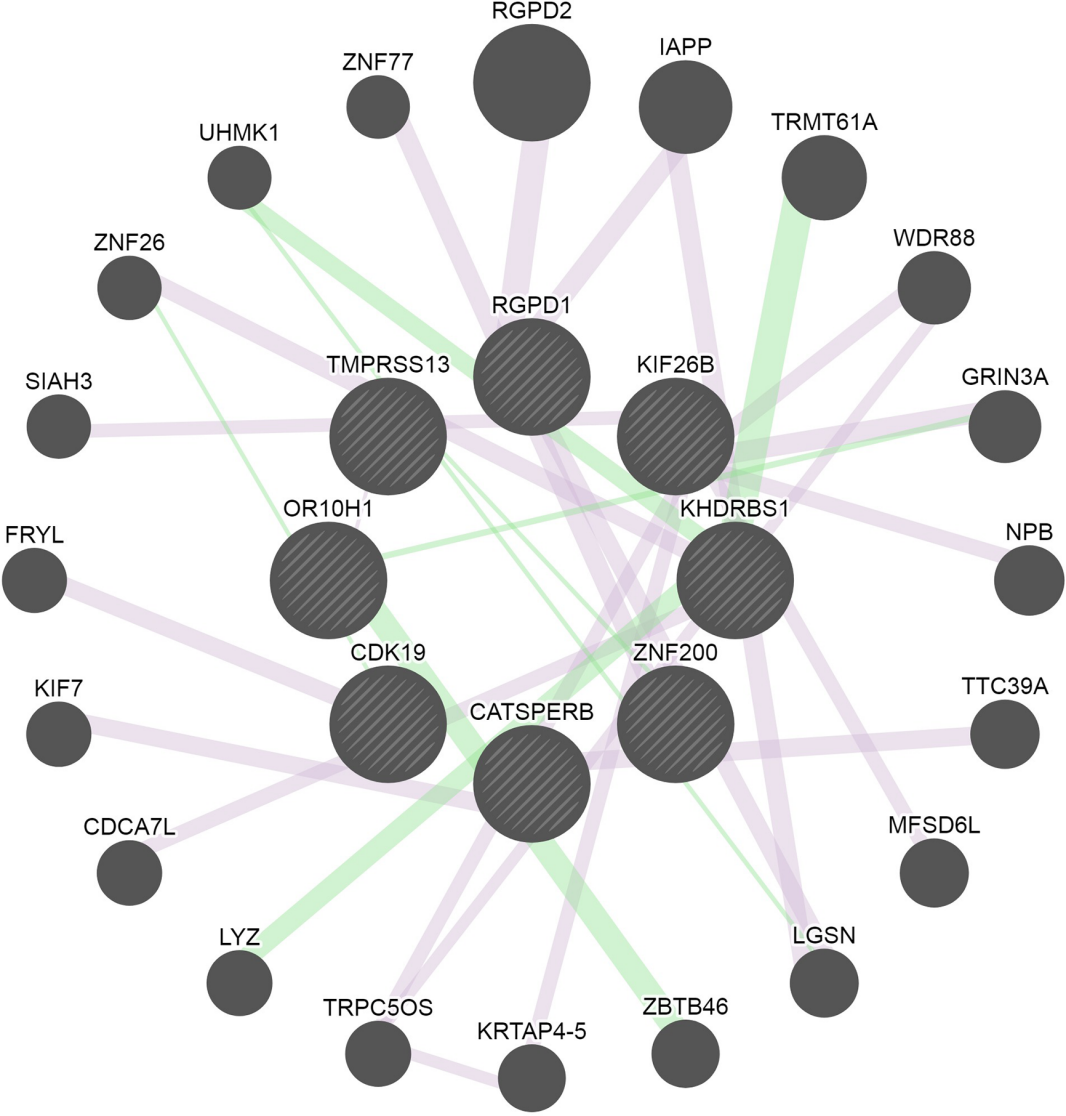
Network of the connected genes identified through GWAS and exome-sequencing analyses. Eight of the identified genes were connected and are represented in the middle circle of the image. Additional genes associated with the genes found in this study are displayed in the outer area of the network circle. The pink lines represent the *physical interaction* reported by several studies. The green lines represent *genetic interactions*. The edges are annotated with the original edge weights (obtained from the source networks and relevant publications). The nodes are annotated with Gene Ontology terms, alternate identifiers and synonyms.

From the genes identified through GWAS and exome-seq analyses, eight genes, including *RGDP1, KIF26B, KHDRBS1, ZNF200, CATSPERB, CDK19, OR10H1* and *TMPRSS13*, were found to be connected in a common network, which show a gene-gene functional interaction with other genes involved in inflammatory responses (**Figure 3**). The pathway analysis using IPA for the 11 genes identified by exome-seq analysis showed enrichment for cellular development (p-value = 3.5 × 10^−4^), molecular transport (p-value= 1.3 × 10^−4^), RNA trafficking (p-value= 1.3 × 10^−4^), cell-to-cell signalling and interaction (p-value= 8.8 × 10^−4^), cellular growth and proliferation (p-value= 8.8 × 10^−4^) (**Supplementary Table S8**). IPA analysis for the four suggestive genes identified through GWAS revealed enrichment for cell morphology (p-value=9.2 × 10^−4^), cellular assembly and organization (p-value= 1.2 × 10^−3^), cellular function and maintenance (p-value= 1.2 × 10^−3^), cell cycle (p-value= 1.3 × 10^−2^) and cellular development (p-value= 2.4 × 10^−2^) (**Supplementary Table S9**). NET-associated genes were mainly involved in cell cycle control of chromosomal replication pathways (p-value= 1.96 × 10^−2^) and molecular cancer pathways (p-value= 1.32 × 10^−1^) (**Supplementary Figure S8**). Diseases linked to the NET-associated genes are cancer (p-value range= 4.91 × 10^−2^ to 7.96 × 10^−4^), organismal injury and abnormalities (p-value range = 4.91 × 10^−2^ to 7.96 × 10^−4^), endocrine system disorders (p-value range= 4.24 × 10^−2^ to 1.81 × 10^−3^), gastrointestinal disease (p-value range= 1.98x10^-2^ – 1.81x10^-3^) and infectious diseases (p-value range= 3.28 × 10^−2^ to 2.10 × 10^−3^).

## Discussion

The present study is the first genetic association study to identify genetic variants and potential loci affecting plasma MPO-DNA complex levels, a marker for NETs. Through GWAS, we found suggestive associations between MPO-DNA complex levels and common SNPs annotated to 4 genes. Through exome-sequencing analysis, we further identified rare exonic variants in 11 genes to be associated with circulating MPO-DNA complex levels. Many of the identified genes are involved in pathways that may be relevant for the function of NETs (e.g., molecular transport and cell-to-cell signalling and interaction). Cancer was the top disease linked to the NET-associated genes found in this study.

In the GWAS analysis, the most significant association was found for a SNP located near a long intergenic non-protein coding RNA 2501 (*LINC02501*). Its function is unknown, and it is yet unclear how this gene contributes to NET formation. We also found one suggestive SNP that was located in the *KIF26B* (Kinesin Family Member 26B) gene. One study that used proteomics identified a protein formed by the *KIF26B* gene that is located on the secretory vesicle membrane of human neutrophils (28). This gene is also associated with different forms of cancer (29, 30). In hepatocellular carcinoma (HCC), *KIF26B* activation was suggested to promote cancer progression by activating the phosphatidylinositol 3-kinase (PI3K) and Akt/Protein Kinase B (PI3K/Akt) signaling pathway (30). The PI3K/Akt signaling pathway is known to be involved in autophagy and has been described as a pathway for NET formation (31). Another interesting suggestive SNP was located in the *CDK19* (Cyclin Dependent Kinase 19) gene. This gene has a role in innate immunity as it regulates NF-κB-mediated gene expression. When stimulated with TNF-alpha, NF-κB heterodimers and CDK19 promote expression of *IL8*, *CXCL2*, and *CXCL3* (32). Different cytokines, including IL-8 have been described to induce NET formation (33). As all these genes were suggestively associated with MPO-DNA complex levels, future studies with larger sample sizes are needed.

In the exome-seq analysis, we found a rare variant in the *TMPRSS13* gene to be associated with MPO-DNA complex levels. This gene is highly expressed in the skin and the mucosa of the esophagus and plays a role in activation of membrane fusion activity of the hemagglutinin (HA) of influenza viruses (34). The family of type II transmembrane serine proteases have been described to play an important role in the HA process. It has also been shown previously that NETs are released in response to several viruses (35, 36). In patients with severe influenza infections, high MPO-DNA complex levels correlate with poor prognosis (37). In contrast, NETs released by mouse neutrophils incubated with Chikungunya virus, resulted in virus capture and neutralization (38). Although NETs may be necessary for the neutralization of specific viruses, the cytotoxic effects of NETs affect survival of the host. From the gene-based analysis, we additionally identified ten genes associated with MPO-DNA complex levels. The gene with the most significant association was *OR10H1* (Olfactory Receptor Family 10 Subfamily H Member 1). *OR10H1* is involved in the olfactory signaling pathway, where adenylyl cyclase mediates cAMP increase and influx of calcium into the cell (39). *OR10H1* is predominantly expressed in the testis and urinary bladder and was shown to be highly expressed in bladder cancer cell lines (40). The direct effect of this gene on NET formation is not yet clear. However, neutrophils from patients with various malignant diseases demonstrate an increased capacity to release NETs (41, 42). This suggests that the association of *OR10H1* with MPO-DNA complex levels could be driven by the presence of malignancy. The second gene identified in exome-seq analysis is the *KHDRBS1* (KH RNA Binding Domain Containing, Signal Transduction) gene. *KHDRBS1* encodes SAM68 (Src-associated-in-mitosis-68-KD), which is an RNA binding protein and Src kinase substrate that has a function in RNA metabolism and signal transduction. It also contributes to TNF-induced NF-κB activation and apoptosis (43) and has been implicated in cancer (44–46). Also, SAM68 is expressed in human neutrophils and is involved in signal transduction when stimulated by phagocytic agonists (47). We also identified one gene that demonstrates an effect in viral transcription and infection, namely *ZNF200* (Zinc Finger Protein 200). This gene exhibits an antiviral function in Herpes simplex virus 1 infection (48). As mentioned earlier, NETs play an important role in viral infections but can be both harmful and beneficial at the same time. Several Zinc Finger Proteins have been identified in activated neutrophils (49). However, *ZNF200* has not yet been shown to be expressed in neutrophils. Moreover, we showed that MPO-DNA complex levels are associated with a set of rare variants annotated to long non-coding RNAs (lncRNAs), namely *RP11-461L13.5, RP11-24B19.4, RP11-461L13.3, RP11-395I6.1, RP11-696P8.2.* It has become increasingly evident that lncRNAs contribute to normal physiology and development of diseases (50). LncRNAs mainly regulate gene expression through modulation of the activity of transcriptional regulators, chromatin remodeling and post-transcriptional modifications (51). The RP-11 lnRNAs we found in our study have not yet been described to be associated with any specific diseases. However, several other RP-11 lncRNAs have previously been shown to play a role in the progression of different types of cancer, including colorectal cancer (52), HCC (53), ovarian cancer (54) and gastric cancer (55). Given cancer was the top disease linked to the NET-associated genes in our pathway analysis, future studies may investigate the potential link between these lncRNAs, NETs and cancer. Also, several lncRNAs have demonstrated to be involved in the regulation of the innate and adaptive immune response (56).

We also found a number of genes that showed a suggestive association with MPO-DNA complex levels in the exome-sequencing analysis, that are worth mentioning. The first gene is *ZDHHC19* (Zinc Finger DHHC-Type Palmitoyltransferase 19), which has a function in inflammation as it regulates STAT3 (57). STAT3 is an important regulator of immunity, inflammation and tumorigenesis (58). Two other suggestive genes we identified in this study, are known to play a role in the PI3K/Akt and mTORC1 (mammalian target of rapamycin activation complex 1) signaling pathway. *FOXC1* (Forkhead Box C1) has been shown to upregulate PI3K/Akt signaling, which was inflammation-dependent in fibroblast-like synoviocytes (59). Also, in HCC cell lines, *FOXC1* promotes inflammation *via* PI3K/Akt signaling (60). In a previous study, both *ZDHHC19* and *FOXC1* were found to be upregulated more than five times in neutrophils from patients with acute respiratory distress syndrome (ARDS) than in neutrophils from healthy volunteers (61). *TMEM55B* (transmembrane protein 55b), a lysosomal protein of unknown molecular function, acts downstream of PI3K/Akt and interacts with many proteins that participate in mTORC1 (62). mTOR suppresses autophagy and has also been reported to be important for NET formation (63).

To our knowledge, this is the first genetic association study that investigated the genetic determinants of NET formation. Our study has several strengths and limitations. The main strength of this study is that we used data from a large population-based study. Moreover, we performed our analysis with both common and rare variants. The gene-based analysis of rare variants was another strength of this study, due to the combined effect of multiple SNPs (19). Furthermore, we measured MPO-DNA complex levels as a marker for the presence of NETs in plasma. MPO-DNA complex is currently considered the most specific, objective and quantitative assay for detecting NETs (64). MPO-DNA complex levels represent the presence of NETs, which are formed through the protein-arginine deiminase type 4 (PAD4)-dependent pathway as well as the autophagy pathway (64). The findings of this study should be considered in light of some limitations. Since this is the first genetic association study for MPO-DNA complex levels, we were unable to replicate our findings in a secondary cohort, which may impede the identification of additional loci. We have included data from European populations; therefore, other studies are needed to assess the generalizability of our findings to other ancestries. Nevertheless, with the results of this study, we have provided potential genetic and molecular pathways leading to NET formation. This may lead to a better understanding of NET-associated diseases. To validate the effect of the genes mentioned in this study, experimental trials and functional experiments are needed.

To conclude, our study found rare variants in 11 genes (*TMPRSS13, OR10H1, RP11-461L13.5, RP11-24B19.4, RP11-461L13.3, KHDRBS1, ZNF200, RP11-395I6.1, RP11-696P8.2, RGPD1, AC007036.5)* that were significantly associated with plasma MPO-DNA complex levels. Moreover, we found suggestive associations between common SNPs annotated to four genes (*KIF26B, CDK19, CATSPERB, AC027119.1*) and MPO-DNA complex levels. These findings may indicate that NET formation is a multifactorial process which is regulated by genes involved in different biological processes. Also, a link between NET-associated genes and cancer was observed. The precise mechanism of how these genes may contribute to neutrophil function or the formation of NETs remains unclear. Future functional studies, investigating the translational potential of these observations is warranted to fully characterized the molecular mechanisms regulated by the genes described in this study.

## Data Availability Statement

The original contributions presented in this study are publicly available. This data can be found here: https://www.ebi.ac.uk/gwas/ accession number: GCST90013658.

## Ethics Statement

The Rotterdam Study was approved by the Medical Ethics Committee of the Erasmus MC and by the Ministry of Health, Welfare and Sport of Netherlands. The patients/participants provided their written informed consent to participate in this study.

## Author Contributions

MG, MD, and FL were responsible for the design and supervision of this project. FR, FV, and MI, collected data. SD and MD were responsible for laboratory measurement of MPO-DNA complex. EP, SA, and MG performed the GWAS, exome sequencing, and *in silico* analyses. SD and EP interpreted the data and contributed equally in writing this article. All authors contributed to the article and approved the submitted version.

## Funding

The Rotterdam Study is supported by the Erasmus MC University Medical Center and Erasmus University Rotterdam; The Netherlands Organisation for Scientific Research (NWO); The Netherlands Organisation for Health Research and Development (ZonMw); the Research Institute for Diseases in the Elderly (RIDE); Netherlands Genomics Initiative (NGI); the Ministry of Education, Culture and Science; the Ministry of Health, Welfare and Sports; the European Commission (DG XII); and the Municipality of Rotterdam. The measurement of MPO-DNA complex levels in participants of the Rotterdam study was supported by a research grant (Prof. Heimburger Award 2018, CSL Behring). We acknowledge the support of the Netherlands Cardiovascular Research Initiative which is supported by the Dutch Heart Foundation (CVON2015-01: CONTRAST), the support of the Brain Foundation Netherlands (HA2015.01.06), and the support of Health~Holland, Top Sector Life Sciences & Health (LSHM17016), Medtronic and Cerenovus. The collaboration project is additionally financed by the Ministry of Economic Affairs by means of the PPP Allowance made available by the Top Sector Life Sciences & Health to stimulate public-private partnerships.

## Conflict of Interest

FL received unrestricted research grants from CSL Behring, Takeda, uniQure, and Sobi for studies unrelated to the presented study. He is a consultant for Takeda, uniQure of which fees are paid to the university and received travel support from Sobi. He is a DSMB member of a study supported by Roche.

The remaining authors declare that the research was conducted in the absence of any commercial or financial relationships that could be construed as a potential conflict of interest.

## References

[1] Brinkmann V Reichard U Goosmann C Fauler B Uhlemann Y Weiss DS. Neutrophil extracellular traps kill bacteria. Science (2004) 303:1532–5. 10.1126/science.1092385 15001782

[2] Fuchs TA Brill A Duerschmied D Schatzberg D Monestier M Myers DD Jr.. Extracellular DNA traps promote thrombosis. Proc Natl Acad Sci U S A (2010) 107:15880–5. 10.1073/pnas.1005743107 PMC2936604 20798043

[3] Autar AS KM Regar E Valgimigli M Leebeek F Zijlstra F De Maat M. Microvascular obstruction in stemi patients during ppci is related to the size of the aspirated coronary artery thrombi and pre-pci neutrophil extracellular trap levels. Eur Heart J (2015) 36(Abstract Supplement):226.

[4] Borissoff JI Joosen IA Versteylen MO Brill A Fuchs TA Savchenko AS. Elevated levels of circulating DNA and chromatin are independently associated with severe coronary atherosclerosis and a prothrombotic state. Arterioscler Thromb Vasc Biol (2013) 33:2032–40. 10.1161/ATVBAHA.113.301627 PMC3806482 23818485

[5] Hakkim A Furnrohr BG Amann K Laube B Abed UA Brinkmann V. Impairment of neutrophil extracellular trap degradation is associated with lupus nephritis. Proc Natl Acad Sci U S A (2010) 107:9813–8. 10.1073/pnas.0909927107 PMC2906830 20439745

[6] Hoppenbrouwers T Boeddha NP Ekinci E Emonts M Hazelzet JA Driessen GJ. Neutrophil extracellular traps in children with meningococcal sepsis. Pediatr Crit Care Med (2018) 19:e286–91. 10.1097/PCC.0000000000001496 29432403

[7] Ikram MA Brusselle G Ghanbari M Goedegebure A Ikram MK Kavousi M. Objectives, design and main findings until 2020 from the rotterdam study. Eur J Epidemiol (2020) 2020:1–35. 10.1007/s10654-020-00640-5 PMC7250962 32367290

[8] McCarthy S Das S Kretzschmar W Delaneau O Wood AR Teumer A. A reference panel of 64,976 haplotypes for genotype imputation. Nat Genet (2016) 48:1279–83. 10.1038/ng.3643 PMC5388176 27548312

[9] Das S Forer L Schönherr S Sidore C Locke AE Kwong A. Next-generation genotype imputation service and methods. Nat Genet (2016) 48:1284–7. 10.1038/ng.3656 PMC5157836 27571263

[10] van Rooij JGJ Jhamai M Arp PP Nouwens SCA Verkerk M Hofman A. Population-specific genetic variation in large sequencing data sets: Why more data is still better. Eur J Hum Genet (2017) 25:1173–5. 10.1038/ejhg.2017.110 PMC5602011 28905877

[11] Grove ML Yu B Cochran BJ Haritunians T Bis JC Taylor KD. Best practices and joint calling of the humanexome beadchip: The charge consortium. PloS One (2013) 8(7):e68095. 10.1371/journal.pone.0068095 23874508 PMC3709915

[12] Wang K Li M Hakonarson H. Annovar: Functional annotation of genetic variants from high-throughput sequencing data. Nucleic Acids Res (2010) 38:e164–4. 10.1093/nar/gkq603 PMC2938201 20601685

[13] Zhan X Hu Y Li B Abecasis GR Liu DJ. Rvtests: An efficient and comprehensive tool for rare variant association analysis using sequence data. Bioinformatics (2016) 32:1423–6. 10.1093/bioinformatics/btw079 PMC4848408 27153000

[14] Winkler TW Day FR Croteau-Chonka DC Wood AR Locke AE Mägi R. Quality control and conduct of genome-wide association meta-analyses. Nat Protoc (2014) 9:1192. 10.1038/nprot.2014.071 24762786 PMC4083217

[15] Willer CJ Li Y Abecasis GR. Metal: Fast and efficient meta-analysis of genomewide association scans. Bioinformatics (2010) 26:2190–1. 10.1093/bioinformatics/btq340 PMC2922887 20616382

[16] de Leeuw CA Mooij JM Heskes T Posthuma D. Magma: Generalized gene-set analysis of gwas data. PloS Comput Biol (2015) 11:e1004219. 10.1371/journal.pcbi.1004219 25885710 PMC4401657

[17] Watanabe K Taskesen E Van Bochoven A Posthuma D. Functional mapping and annotation of genetic associations with fuma. Nat Commun (2017) 8:1–11. 10.1038/s41467-017-01261-5 29184056 PMC5705698

[18] Mathieson I McVean G. Differential confounding of rare and common variants in spatially structured populations. Nat Genet (2012) 44:243. 10.1038/ng.1074 22306651 PMC3303124

[19] Voorman A Brody J Chen H Lumley T. Seqmeta: An r package for meta-analyzing region-based tests of rare DNA variants. R Package Version (2013) 1:3.

[20] Bulik-Sullivan BK Loh P-R Finucane HK Ripke S Yang J Patterson N. Ld score regression distinguishes confounding from polygenicity in genome-wide association studies. Nat Genet (2015) 47:291. 10.1038/ng.3211 25642630 PMC4495769

[21] Pasaniuc B Price AL. Dissecting the genetics of complex traits using summary association statistics. Nat Rev Genet (2017) 18:117–27. 10.1038/nrg.2016.142 PMC5449190 27840428

[22] Bulik-Sullivan B Finucane HK Anttila V Gusev A Day FR Loh P-R. An atlas of genetic correlations across human diseases and traits. Nat Genet (2015) 47:1236. 10.1038/ng.3406 26414676 PMC4797329

[23] Ward LD Kellis M. Haploreg: A resource for exploring chromatin states, conservation, and regulatory motif alterations within sets of genetically linked variants. Nucleic Acids Res (2012) 40:D930–4. 10.1093/nar/gkr917 PMC3245002 22064851

[24] Welter D MacArthur J Morales J Burdett T Hall P Junkins H. The nhgri gwas catalog, a curated resource of snp-trait associations. Nucleic Acids Res (2014) 42:D1001–6. 10.1093/nar/gkt1229 PMC3965119 24316577

[25] Lonsdale J Thomas J Salvatore M Phillips R Lo E Shad S. The genotype-tissue expression (gtex) project. Nat Genet (2013) 45(6):580–5. 10.1038/ng.2653 PMC4010069 23715323

[26] Warde-Farley D Donaldson SL Comes O Zuberi K Badrawi R Chao P. The genemania prediction server: Biological network integration for gene prioritization and predicting gene function. Nucleic Acids Res (2010) 38:W214–20. 10.1093/nar/gkq537 PMC2896186 20576703

[27] Canela-Xandri O Rawlik K Tenesa A. An atlas of genetic associations in uk biobank. Nat Genet (2018) 50:1593–9. 10.1038/s41588-018-0248-z PMC6707814 30349118

[28] Uriarte SM Powell DW Luerman GC Merchant ML Cummins TD Jog NR. Comparison of proteins expressed on secretory vesicle membranes and plasma membranes of human neutrophils. J Immunol (2008) 180:5575–81. 10.4049/jimmunol.180.8.5575 18390742

[29] Li H Shen S Chen X Ren Z Li Z Yu Z. Mir-450b-5p loss mediated kif26b activation promoted hepatocellular carcinoma progression by activating pi3k/akt pathway. Cancer Cell Int (2019) 19:205. 10.1186/s12935-019-0923-x 31388332 PMC6670205

[30] Teng Y Guo B Mu X Liu S. Kif26b promotes cell proliferation and migration through the fgf2/erk signaling pathway in breast cancer. BioMed Pharmacother (2018) 108:766–73. 10.1016/j.biopha.2018.09.036 30248545

[31] Remijsen Q Vanden Berghe T Wirawan E Asselbergh B Parthoens E De Rycke R. Neutrophil extracellular trap cell death requires both autophagy and superoxide generation. Cell Res (2011) 21:290–304. 10.1038/cr.2010.150 21060338 PMC3193439

[32] Chen M Liang J Ji H Yang Z Altilia S Hu B. Cdk8/19 mediator kinases potentiate induction of transcription by nfkappab. Proc Natl Acad Sci U S A (2017) 114:10208–13. 10.1073/pnas.1710467114 PMC5617299 28855340

[33] Hoppenbrouwers T Autar ASA Sultan AR Abraham TE van Cappellen WA Houtsmuller AB. In vitro induction of netosis: Comprehensive live imaging comparison and systematic review. PloS One (2017) 12:e0176472. 10.1371/journal.pone.0176472 28486563 PMC5423591

[34] Okumura Y Takahashi E Yano M Ohuchi M Daidoji T Nakaya T. Novel type ii transmembrane serine proteases, mspl and tmprss13, proteolytically activate membrane fusion activity of the hemagglutinin of highly pathogenic avian influenza viruses and induce their multicycle replication. J Virol (2010) 84:5089–96. 10.1128/JVI.02605-09 PMC2863848 20219906

[35] Funchal GA Jaeger N Czepielewski RS Machado MS Muraro SP Stein RT. Respiratory syncytial virus fusion protein promotes tlr-4-dependent neutrophil extracellular trap formation by human neutrophils. PloS One (2015) 10:e0124082. 10.1371/journal.pone.0124082 25856628 PMC4391750

[36] Saitoh T Komano J Saitoh Y Misawa T Takahama M Kozaki T. Neutrophil extracellular traps mediate a host defense response to human immunodeficiency virus-1. Cell Host Microbe (2012) 12:109–16. 10.1016/j.chom.2012.05.015 22817992

[37] Zhu L Liu L Zhang Y Pu L Liu J Li X. High level of neutrophil extracellular traps correlates with poor prognosis of severe influenza a infection. J Infect Dis (2018) 217:428–37. 10.1093/infdis/jix475 29325098

[38] Hiroki CH Toller-Kawahisa JE Fumagalli MJ Colon DF Figueiredo LTM Fonseca B. Neutrophil extracellular traps effectively control acute chikungunya virus infection. Front Immunol (2019) 10:3108. 10.3389/fimmu.2019.03108 32082301 PMC7005923

[39] Bakalyar HA Reed RR. The second messenger cascade in olfactory receptor neurons. Curr Opin Neurobiol (1991) 1:204–8. 10.1016/0959-4388(91)90079-M 1668220

[40] Weber L Schulz WA Philippou S Eckardt J Ubrig B Hoffmann MJ. Characterization of the olfactory receptor or10h1 in human urinary bladder cancer. Front Physiol (2018) 9:456. 10.3389/fphys.2018.00456 29867524 PMC5964926

[41] Podaza E Sabbione F Risnik D Borge M Almejun MB Colado A. Neutrophils from chronic lymphocytic leukemia patients exhibit an increased capacity to release extracellular traps (nets). Cancer Immunol Immunother (2017) 66:77–89. 10.1007/s00262-016-1921-7 27796477 PMC11029506

[42] Rayes RF Mouhanna JG Nicolau I Bourdeau F Giannias B Rousseau S. Primary tumors induce neutrophil extracellular traps with targetable metastasis promoting effects. JCI Insight (2019) 5(16):e128008. 10.1172/jci.insight.128008 PMC6777835 31343990

[43] Ramakrishnan P Baltimore D. Sam68 is required for both nf-kappab activation and apoptosis signaling by the tnf receptor. Mol Cell (2011) 43:167–79. 10.1016/j.molcel.2011.05.007 PMC3142289 21620750

[44] Fu K Sun X Wier EM Hodgson A Liu Y Sears CL. Sam68/khdrbs1 is critical for colon tumorigenesis by regulating genotoxic stress-induced nf-kappab activation. Elife (2016) 5:e15018. 10.7554/eLife.15018 27458801 PMC4959885

[45] Busa R Paronetto MP Farini D Pierantozzi E Botti F Angelini DF. The rna-binding protein sam68 contributes to proliferation and survival of human prostate cancer cells. Oncogene (2007) 26:4372–82. 10.1038/sj.onc.1210224 17237817

[46] Zhang Z Li J Zheng H Yu C Chen J Liu Z. Expression and cytoplasmic localization of sam68 is a significant and independent prognostic marker for renal cell carcinoma. Cancer Epidemiol Biomarkers Prev (2009) 18:2685–93. 10.1158/1055-9965.EPI-09-0097 19755649

[47] Gilbert C Barabe F Rollet-Labelle E Bourgoin SG McColl SR Damaj BB. Evidence for a role for sam68 in the responses of human neutrophils to ligation of cd32 and to monosodium urate crystals. J Immunol (2001) 166:4664–71. 10.4049/jimmunol.166.7.4664 11254726

[48] Bick MJ Carroll JW Gao G Goff SP Rice CM MacDonald MR. Expression of the zinc-finger antiviral protein inhibits alphavirus replication. J Virol (2003) 77:11555–62. 10.1128/JVI.77.21.11555-11562.2003 PMC229374 14557641

[49] Zhang X Kluger Y Nakayama Y Poddar R Whitney C DeTora A. Gene expression in mature neutrophils: Early responses to inflammatory stimuli. J Leukoc Biol (2004) 75:358–72. 10.1189/jlb.0903412 14634056

[50] Esteller M. Non-coding rnas in human disease. Nat Rev Genet (2011) 12:861–74. 10.1038/nrg3074 22094949

[51] Kopp F Mendell JT. Functional classification and experimental dissection of long noncoding rnas. Cell (2018) 172:393–407. 10.1016/j.cell.2018.01.011 29373828 PMC5978744

[52] Wu Y Yang X Chen Z Tian L Jiang G Chen F. M(6)a-induced lncrna rp11 triggers the dissemination of colorectal cancer cells via upregulation of zeb1. Mol Cancer (2019) 18:87. 10.1186/s12943-019-1014-2 30979372 PMC6461827

[53] Zhang J Zhang D Zhao Q Qi J Li X Qin C. A distinctively expressed long noncoding rna, rp11-466i1.1, may serve as a prognostic biomarker in hepatocellular carcinoma. Cancer Med (2018) 7(7):2960–8. 10.1002/cam4.1565 PMC6051177 29790663

[54] Huang K Geng J Wang J. Long non-coding rna rp11-552m11.4 promotes cells proliferation, migration and invasion by targeting brca2 in ovarian cancer. Cancer Sci (2018) 109:1428–46. 10.1111/cas.13552 PMC5980309 29478268

[55] Chen FR Sha SM Wang SH Shi HT Dong L Liu D. Rp11-81h3.2 promotes gastric cancer progression through mir-339-hnrnpa1 interaction network. Cancer Med (2020) 9:2524–34. 10.1002/cam4.2867 PMC7131847 32052594

[56] Mumtaz PT Bhat SA Ahmad SM Dar MA Ahmed R Urwat U. Lncrnas and immunity: Watchdogs for host pathogen interactions. Biol Proced Online (2017) 19:3. 10.1186/s12575-017-0052-7 28465674 PMC5406993

[57] Niu J Sun Y Chen B Zheng B Jarugumilli GK Walker SR. Fatty acids and cancer-amplified zdhhc19 promote stat3 activation through s-palmitoylation. Nature (2019) 573:139–43. 10.1038/s41586-019-1511-x PMC6728214 31462771

[58] Yu H Lee H Herrmann A Buettner R Jove R. Revisiting stat3 signalling in cancer: New and unexpected biological functions. Nat Rev Cancer (2014) 14:736–46. 10.1038/nrc3818 25342631

[59] Yu Z Xu H Wang H Wang Y. Foxc1 promotes the proliferation of fibroblast-like synoviocytes in rheumatoid arthritis via pi3k/akt signalling pathway. Tissue Cell (2018) 53:15–22. 10.1016/j.tice.2018.05.011 30060822

[60] Huang W Chen Z Zhang L Tian D Wang D Fan D. Interleukin-8 induces expression of foxc1 to promote transactivation of cxcr1 and ccl2 in hepatocellular carcinoma cell lines and formation of metastases in mice. Gastroenterology (2015) 149:1053–67.e1014. 10.1053/j.gastro.2015.05.058 26065367

[61] Juss JK House D Amour A Begg M Herre J Storisteanu DM. Acute respiratory distress syndrome neutrophils have a distinct phenotype and are resistant to phosphoinositide 3-kinase inhibition. Am J Respir Crit Care Med (2016) 194:961–73. 10.1164/rccm.201509-1818OC PMC5067816 27064380

[62] Hashimoto Y Shirane M Nakayama KI. Tmem55b contributes to lysosomal homeostasis and amino acid-induced mtorc1 activation. Genes Cells (2018) 23:418–34. 10.1111/gtc.12583 29644770

[63] McInturff AM Cody MJ Elliott EA Glenn JW Rowley JW Rondina MT. Mammalian target of rapamycin regulates neutrophil extracellular trap formation via induction of hypoxia-inducible factor 1 alpha. Blood (2012) 120:3118–25. 10.1182/blood-2012-01-405993 PMC3471519 22919032

[64] Masuda S Nakazawa D Shida H Miyoshi A Kusunoki Y Tomaru U. Netosis markers: Quest for specific, objective, and quantitative markers. Clin Chim Acta (2016) 459:89–93. 10.1016/j.cca.2016.05.029 27259468

