# Supplementary material for: Common and Rare Variants Genetic Association Analysis of Circulating Neutrophil Extracellular Traps
Source: Front Immunol. 2021 Feb 24;12:615527. doi: 10.3389/fimmu.2021.615527 (PMC7944992; doi:10.3389/fimmu.2021.615527)
Supplement: Supplementary file 1 [file DataSheet_1.pdf]

Figure S1. Quantile-quantile plot of genome-wide association studies on MPO-DNA complex levels

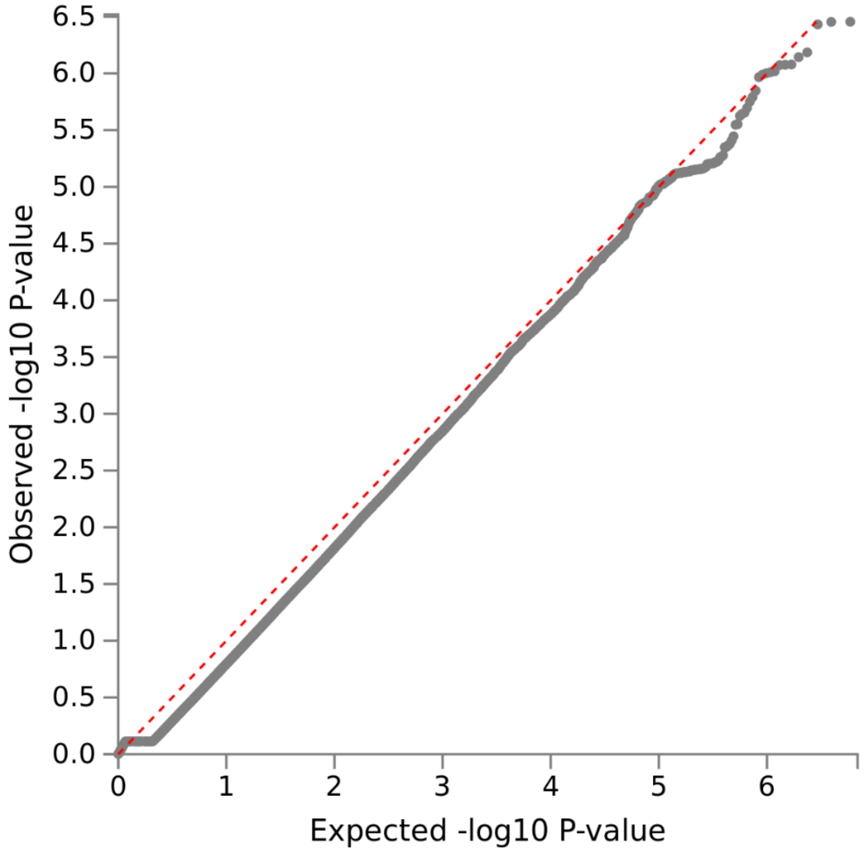

Vertical and horizontal lines represent expected p-values under a null distribution and observed p-values, respectively. Lambda=1.02

Figure S2: Gene expression for *OR10H1*

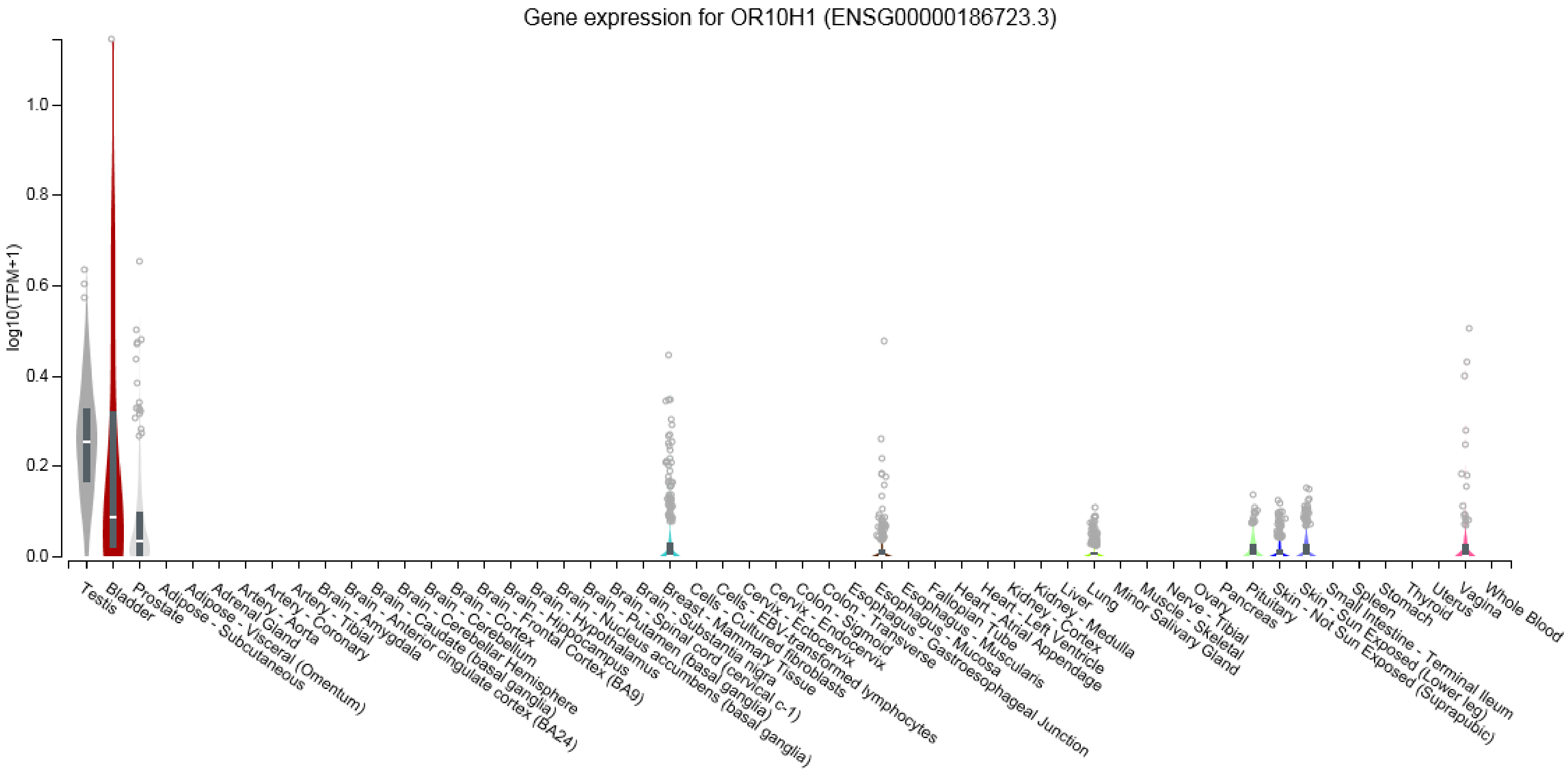

Gene expression in different tissues. The gene expression values are shown in TPM (transcripts per million), calculated from a gene model with isoforms collapsed to a single gene. Box plots are shown as median and 25th and 75th percentiles, outliers are displayed as dots if they are above or below 1.5 times the interquartile range.

Figure S3: Gene expression for *RP11-461L13.5*

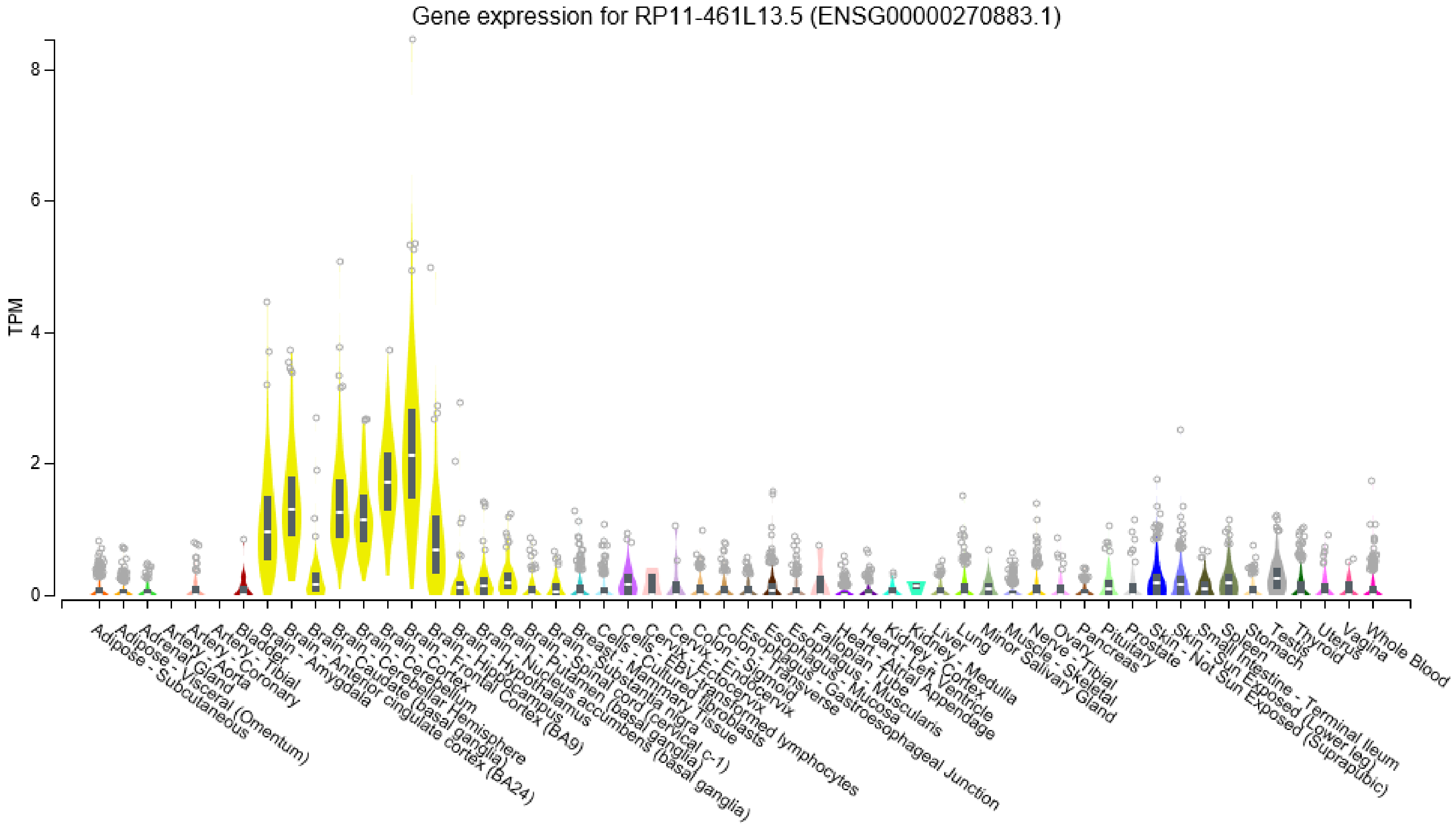

Gene expression in different tissues. The gene expression values are shown in TPM (transcripts per million), calculated from a gene model with isoforms collapsed to a single gene. Box plots are shown as median and 25th and 75th percentiles, outliers are displayed as dots if they are above or below 1.5 times the interquartile range.

Figure S4: Gene expression for *RP11-461L13.3*

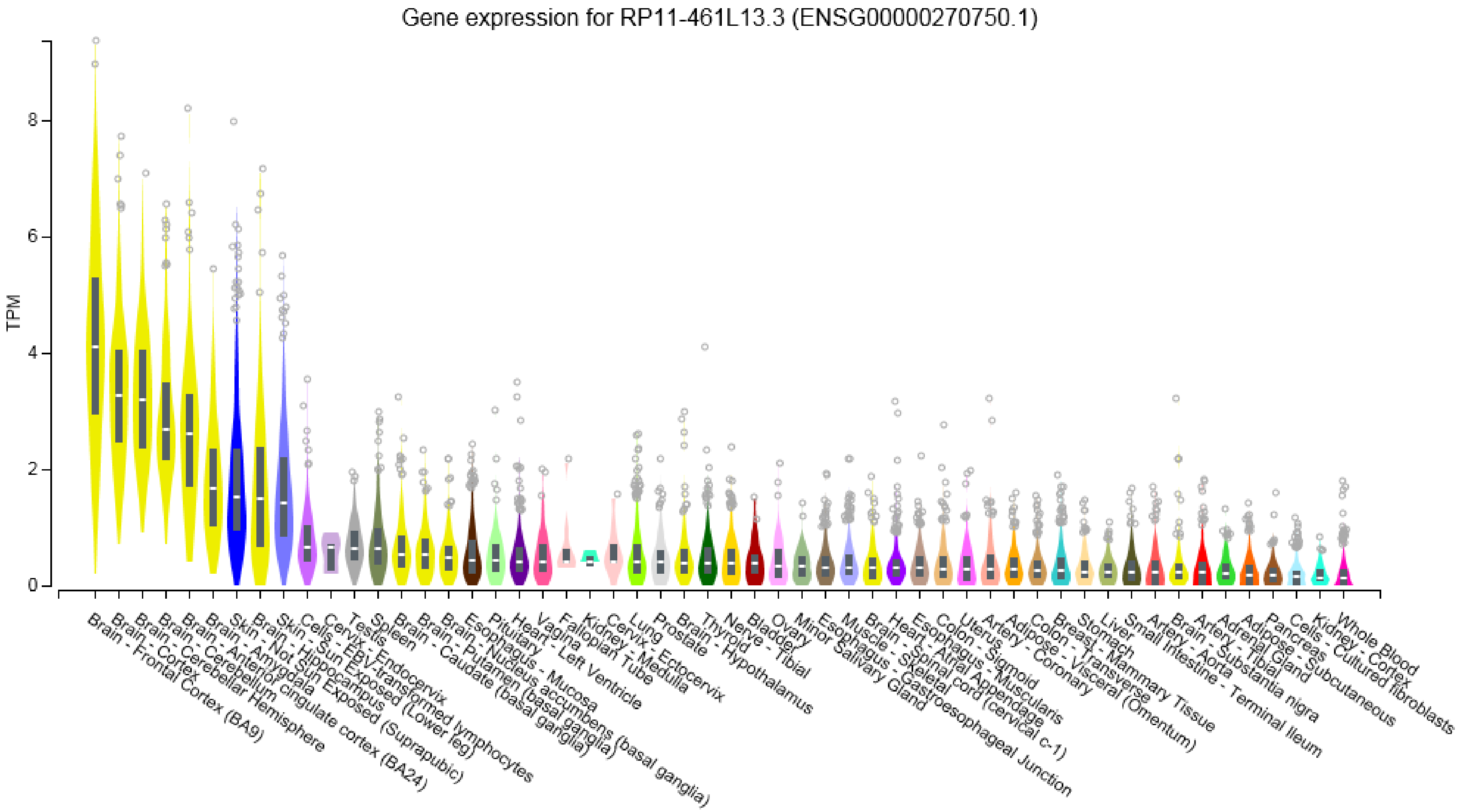

Gene expression in different tissues. The gene expression values are shown in TPM (transcripts per million), calculated from a gene model with isoforms collapsed to a single gene. Box plots are shown as median and 25th and 75th percentiles, outliers are displayed as dots if they are above or below 1.5 times the interquartile range.

Figure S5: Gene expression for *KHDRBS1*

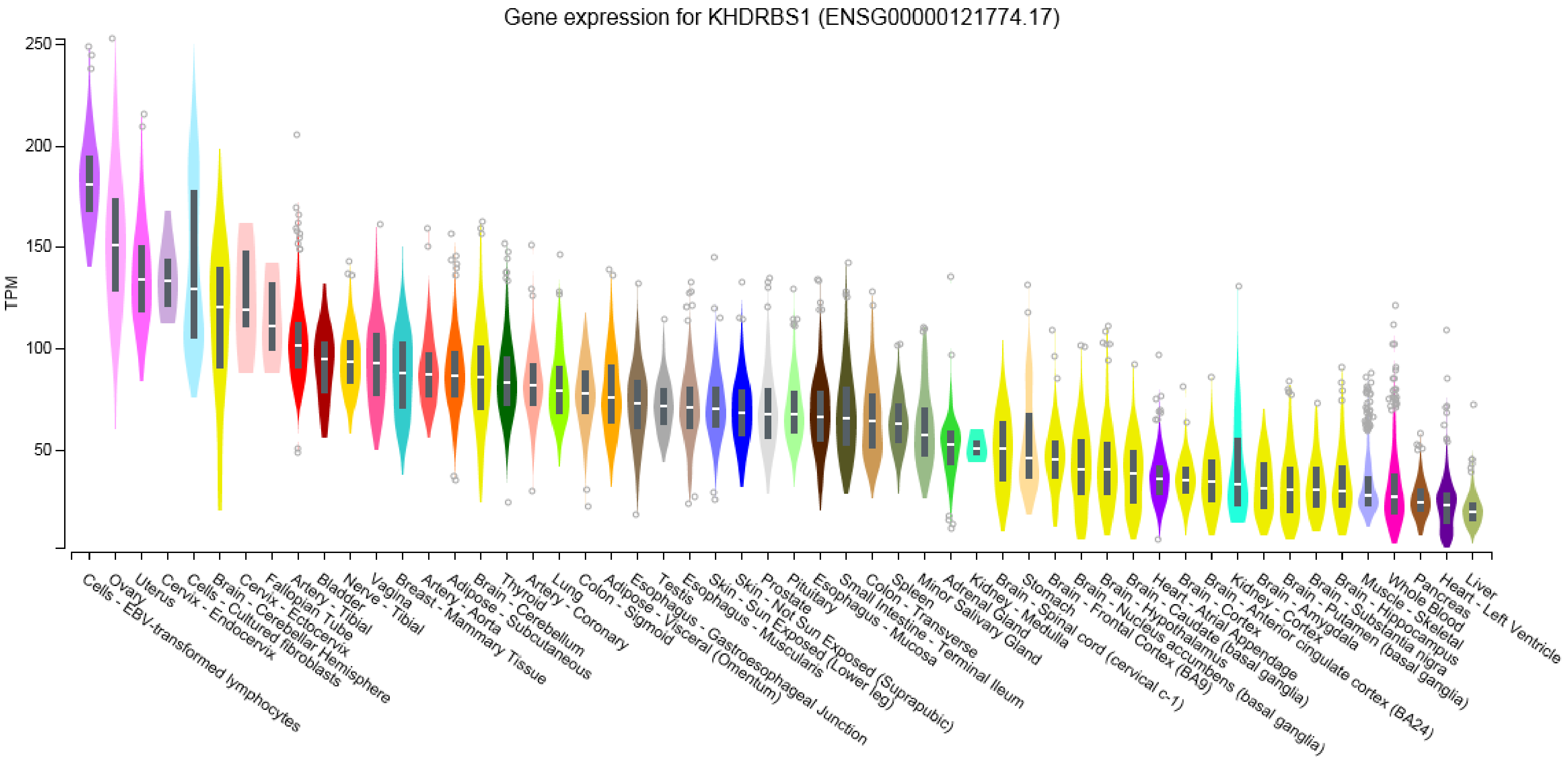

Gene expression in different tissues. The gene expression values are shown in TPM (transcripts per million), calculated from a gene model with isoforms collapsed to a single gene. Box plots are shown as median and 25th and 75th percentiles, outliers are displayed as dots if they are above or below 1.5 times the interquartile range.

Figure S6: Gene expression for *ZNF200*

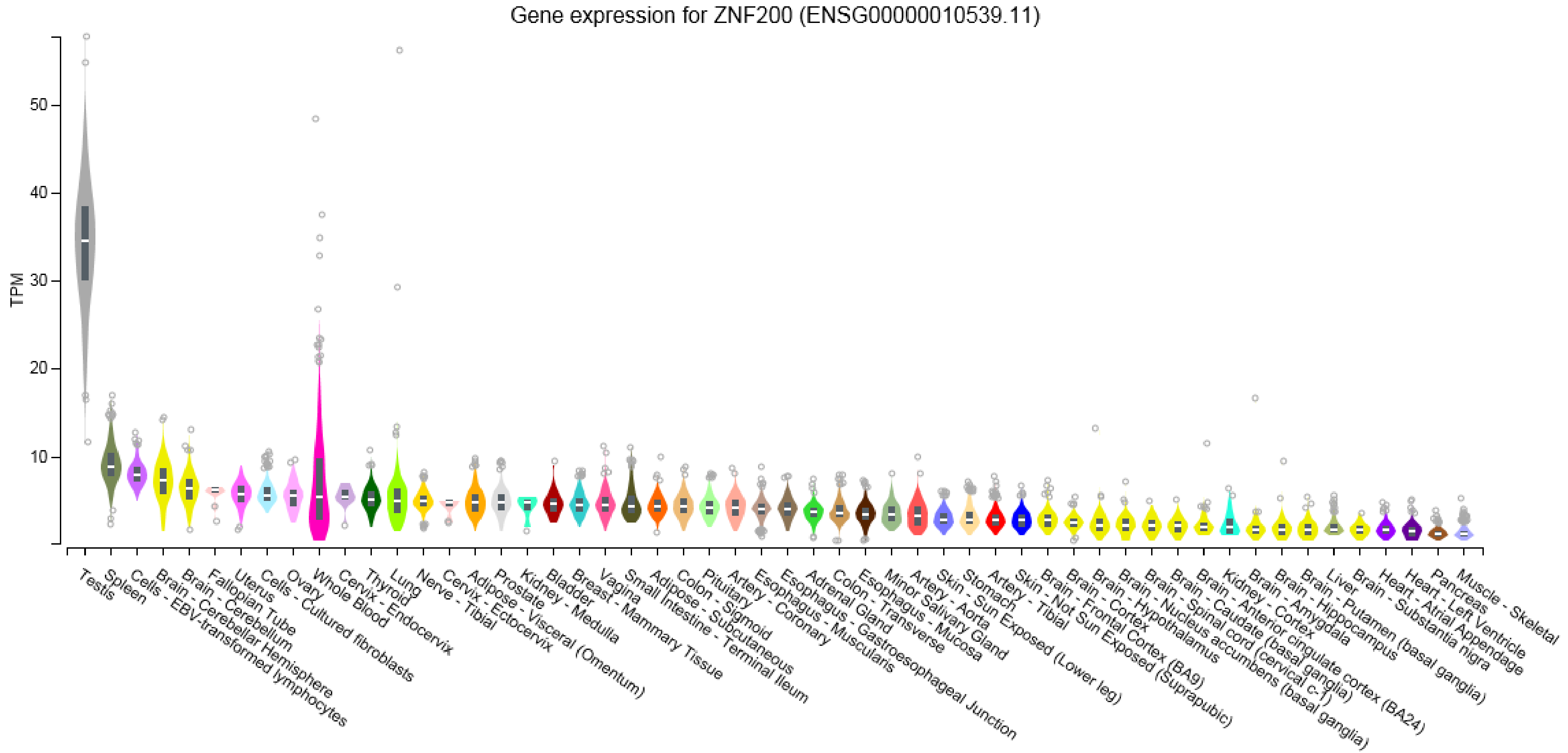

Gene expression in different tissues. The gene expression values are shown in TPM (transcripts per million), calculated from a gene model with isoforms collapsed to a single gene. Box plots are shown as median and 25th and 75th percentiles, outliers are displayed as dots if they are above or below 1.5 times the interquartile range.

Figure S7: Gene expression for *RGPD1*

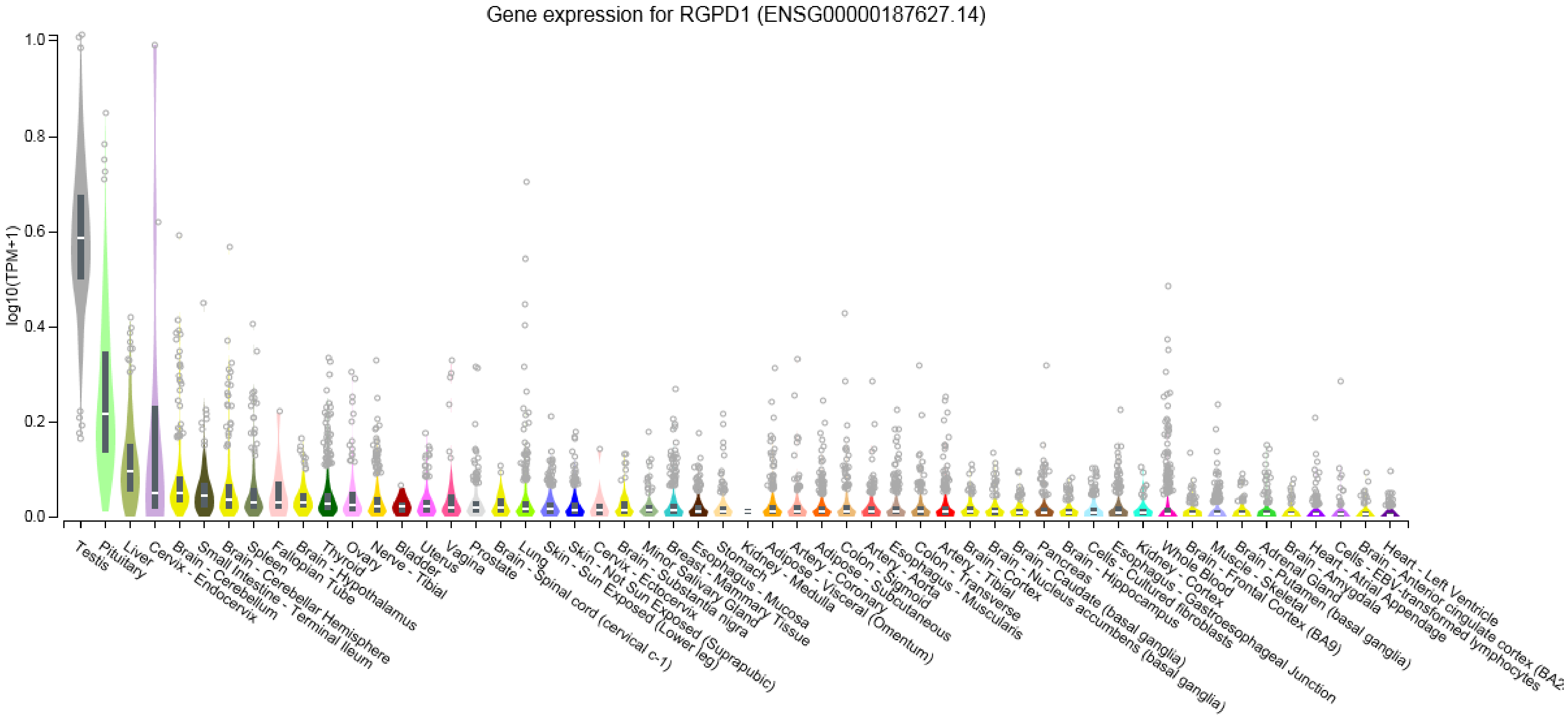

Gene expression in different tissues. The gene expression values are shown in TPM (transcripts per million), calculated from a gene model with isoforms collapsed to a single gene. Box plots are shown as median and 25th and 75th percentiles, outliers are displayed as dots if they are above or below 1.5 times the interquartile range.

Figure S8. NET-associated genes involved in cancer pathways.

Path Designer Network 1

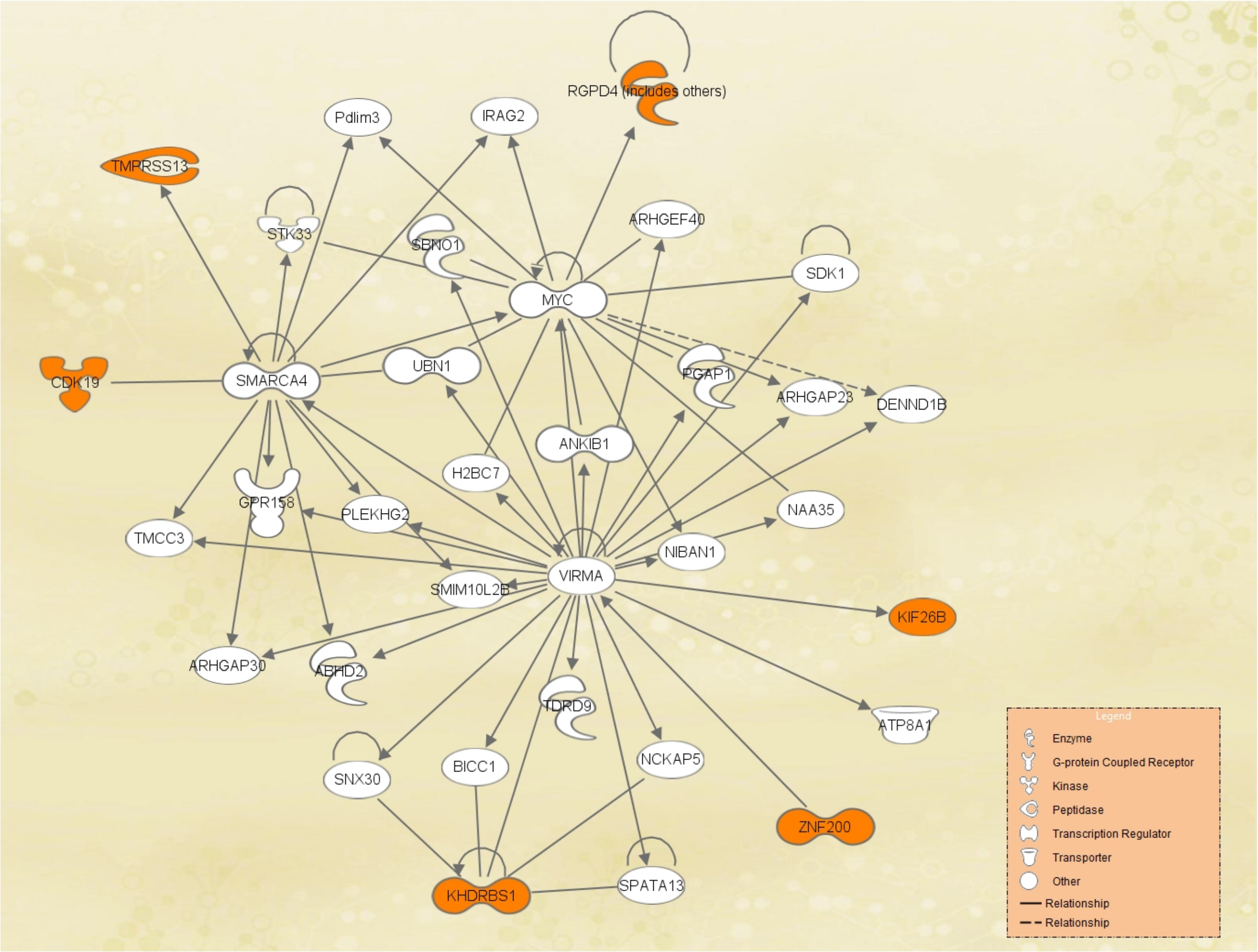

© 2000-2020 QIAGEN. All rights reserved.

The identified NET-associated genes are indicated in orange and are associated with genes involved in cancer pathways.
